# Pressure-Induced Structural Phase Transition in Ho_2_Ce_2_O_7_ Oxide

**DOI:** 10.3390/ma18122729

**Published:** 2025-06-10

**Authors:** Tao Lv, Jia Qv, Limin Yan, Yan Li, Qiang Tao, Pinwen Zhu, Xin Wang

**Affiliations:** 1 State Key Laboratory of High Pressure and Superhard Materials, Jilin University, Changchun 130012, China; 2 College of Physics, Jilin University, Changchun 130012, China

**Keywords:** Ho_2_Ce_2_O_7_, structural phase transition, the rare earth C-type structure, high pressure

## Abstract

The structural evolution of Ho_2_Ce_2_O_7_ under high pressure was systematically investigated using synchrotron X-ray diffraction (up to 31.5 GPa) and Raman spectroscopy (up to 41.7 GPa). At ambient pressure, the compound adopts a common C-type cubic rare earth oxide structure (space group *Ia-3*). A pressure-induced phase transition was observed to commence at 23.8 GPa, characterized by a gradual structural evolution that persisted through the maximum experimental pressure of 31.5 GPa. This transition involves cation disordering accompanied by coordination environment modifications. High-pressure X-ray diffraction analysis reveals the coexistence of two distinct phases above the transition threshold: the parent cubic phase (*Ia-3*) and a metastable hexagonal phase (*R-3c*). Notably, the high-pressure phase configuration persists upon complete decompression to ambient conditions, demonstrating the irreversible nature of this pressure-induced structural transition.

## 1. Introduction

Rare earth-doped cerium oxide compounds have attracted significant interest due to their remarkable proton conductivity, low thermal conductivity, good mechanical properties and high thermal expansion coefficients [1,2,3,4,5,6,7,8,9]. Among these, RE_2_Ce_2_O_7_ (RE = rare earth elements) has gained attention as a promising candidate for applications in solid oxide fuel cells, photocatalysis and electrochemical hydrogen storage [10,11,12,13,14,15,16,17]. According to M.A. Subramanian et al. [18], the disorder of A_2_B_2_O_7_ solid solution where A is a trivalent lanthanide and B is a tetravalent cation is critically governed by the ionic radius ratio of A^3+^ and B^4+^ cations. The pyrochlore structure remains stable when 1.46 ≤ r(A^3+^)/r(B^4+^) ≤ 1.78, while a lower ratio of r(A^3+^)/r(B^4+^) results in the formation of a disordered fluorite structure (if it is disordered). Yamamura et al. [19] suggests that a fluorite (F-type) structure (accompanying the rare earth C-type superstructure) will form for RE_2_Ce_2_O_7_ when the ionic radius ratio of r(RE^3+^)/r(Ce^4+^) is lower than 1.17. For instance, Ho_2_Ce_2_O_7_ has garnered significant attention due to its high oxygen vacancies and proton conductivity, but its crystal structure is poorly understood. Since the tolerance factor (τ) of Ho_2_Ce_2_O_7_ is 0.97, which is slightly less than 1.17, it would be considered to have a disordered fluorite structure (accompanying the rare earth C-type superstructure).

As is well known, pressure is a powerful tool to modify the structure and properties of various materials. Under high pressure, the pyrochlore-type Nd_2_Ir_2_O_7_ oxide was reported to undergo a pressure-induced magnetic phase transition [20]. The pressure-tuned insulator-metal transition was found in Eu_2_Ir_2_O_7_ [21]. The photoluminescence properties of Ho_2_Sn_2_O_7_ were significantly enhanced as a result of the structure phase transition induced by pressure treatment [22]. The fluorite-type La_2_Ce_2_O_7_ oxide can occur via structural phase transition by varying temperature or pressure [23]. These extreme experimental conditions can be used to effectively change the crystal structure, including changing the interatomic distance and polyhedral distortion. At ambient pressure, RE_2_Ce_2_O_7_ oxides exhibit notable proton conductivity owing to their unique crystal structure with abundant oxygen vacancies [3,10,11,24,25]. We propose a pressure-induced structural engineering strategy to enhance oxygen vacancy density in the RE_2_Ce_2_O_7_ oxide, thereby establishing optimized proton migration channels and significantly improving the protonic conduction efficiency within this material system.

Previous studies on Ho_2_O_3_-doped CeO_2_ solid solutions primarily focused on the structures under different doping ratios [26]. However, the structural evolution of Ho_2_Ce_2_O_7_ under high pressure remains largely unexplored. In this study, we employ in situ synchrotron X-ray diffraction and Raman spectroscopy to investigate the structural and optical evolution of Ho_2_Ce_2_O_7_ under hydrostatic compression up to 31.5 GPa and 41.7 GPa. Our results reveal a phase transition in Ho_2_Ce_2_O_7_ occurring near 23.8 GPa. This transition involves cation disordering accompanied by coordination environment modifications. Furthermore, the high-pressure phase remains stable even after complete decompression, providing compelling evidence for an irreversible structural phase transition. Investigating the pressure-induced structural evolution and optical response in Ho_2_Ce_2_O_7_ oxide provides crucial insights for rational design of advanced functional materials with remarkable proton conductivity.

## 2. Materials and Methods

A high-quality Ho_2_Ce_2_O_7_ sample was synthesized by solid-state reaction with the raw materials Ho_2_O_3_ (99.99%) and CeO_2_ (99.99%) [23]. The raw materials were thoroughly mixed in a stoichiometric ratio (Ho_2_O_3_: CeO_2_ = 1:2) and calcined at 1673 K for 12 h. High-pressure experiments were performed using a symmetric diamond anvil cell (DAC) equipped with 400 μm diamond culet anvils. The sample was loaded into a 120 μm diameter hole that was drilled in the center of the T301 stainless steel gasket with a pre-indented thickness of 50 μm. The pressure was calibrated using the standard ruby fluorescent technique [27]. Silicone oil was employed as a pressure-transmitting medium to provide quasi-hydrostatic pressure conditions [28]. High-pressure experiments were conducted under ambient temperature.

In situ high-pressure angle-dispersive X-ray diffraction (ADXRD) measurements up to 31.5 GPa were carried out at a beam line of BL04-MSPD in the ALBA synchrotron light facility with monochromatic X-ray beam (λ = 0.4246 Å). The monochromatic X-ray beam with a wavelength of 0.4246 Å was focused to a spot size of approximately 20 × 20 µm (full width at half maximum (FWHM)). Diffraction data were collected using a Rayonix SX165 charge-coupled device (CCD) detector. For instrument calibration, high-purity LaB_6_ powder was employed as the standard to determine the sample-to-detector distance. DIOPTAS 0.5.7 software was used to integrate the two-dimensional diffraction patterns to one-dimensional intensity versus two theta plots. The Rietveld method was employed to analyze the structure model of diffraction patterns; the GSAS program was utilized to refine ADXRD profiles. High-pressure Raman scattering measurements were conducted up to 41.3 GPa, utilizing a micro-Raman spectrometer (Renishaw) with a 532 nm excitation laser. The Raman signals were recorded in backscattering geometry through a triple polychromator spectrometer equipped with a volume transmission grating, and the laser power was maintained at 50 mW. Sample irradiation was achieved using an Olympus objective lens (20.5 mm focal length, 0.35 numerical aperture) to focus the laser beam onto the sample surface. The Raman spectral peaks were deconvoluted using the Gaussian fitting function in OriginPro 2017 software, with their characteristic positions calibrated against standard reference spectra [29,30].

## 3. Results and Discussion

**Figure 1:**
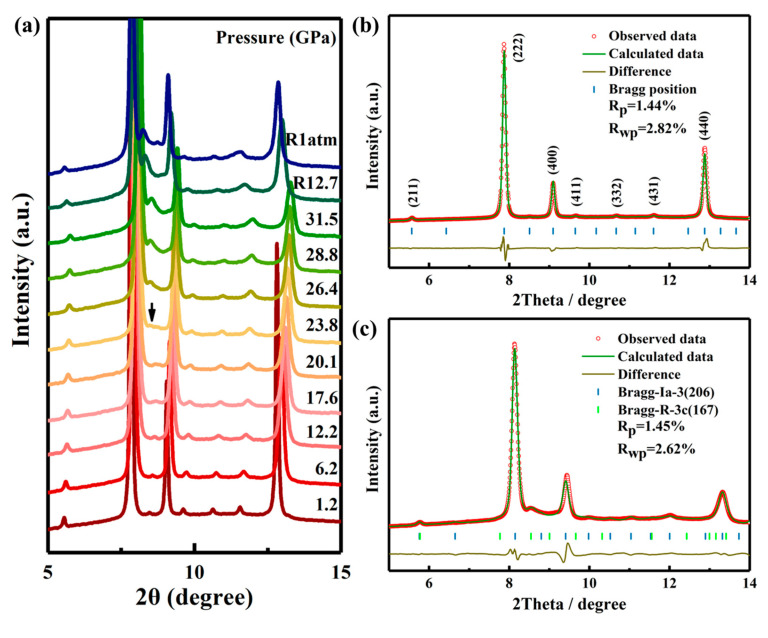
(**a**) Representative ADXRD patterns of Ho_2_Ce_2_O_7_ at various pressures. (**b**,**c**) show the Rietveld refinements of ADXRD patterns collected at 1.2 and 28.8 GPa, respectively. The brown lines denote the difference between the observed (red circles) and calculated (green lines) profiles, and the blue and green verticals stand for the simulated peak positions.

To investigate the structural stability of Ho_2_Ce_2_O_7_ under high-pressure conditions, a systematic study was conducted using synchrotron X-ray diffraction. Figure 1a shows the selected ADXRD patterns of Ho_2_Ce_2_O_7_ up to 31.5 GPa. At lower pressures, all diffraction peaks can be indexed to a C-type rare earth structure. As the pressure increases, all diffraction peaks shift to higher 2θ values (no the initial peaks disappear or new peaks appear). The broadening and weakening of peaks in Ho_2_Ce_2_O_7_ may be caused by less hydrostatic condition at pressures above 12 GPa when there is a glass transition of the pressure-transmitting medium silicone oil [28]. At 23.8 GPa, a structural phase transition was occurring with the appearance of an additional diffraction peak (~8.5°), which is the strongest diffraction peak from the high-pressure phase. Meanwhile, the new diffraction peak gradually became stronger with pressure. The pressure-induced phase transition in Ho_2_Ce_2_O_7_ is sluggish, similar to those observed in other pyrochlore or fluorite-structured oxides [23,31]. Remarkably, the initial crystalline phase remained predominant throughout the compression cycle, suggesting that the transformation remained incomplete at the maximum applied pressure of 31.5 GPa. A previous investigation demonstrated that the fluorite-type structure in La_2_Ce_2_O_7_ undergoes a partly reversible pressure-induced phase transition [23]. However, the high-pressure phase of Ho_2_Ce_2_O_7_ was retained after fully releasing pressure, which indicates that the structural phase transition was irreversible.

**Figure 2:**
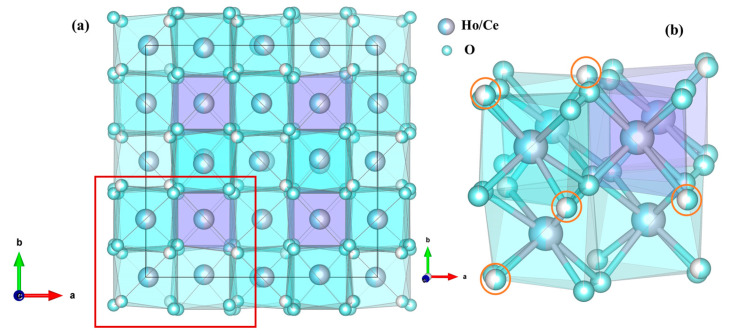
(**a**) The corresponding crystal structure of the cubic Ho_2_Ce_2_O_7_. (**b**) A magnified view of the marked region.

In order to obtain more information about the phase transition, high-pressure structural evolution of Ho_2_Ce_2_O_7_ was determined by Rietveld refinement of the ADXRD pattern via GSAS. As shown in Figure 1b, the good refinement between the observed pattern and the calculated result (R_p_ = 1.44%, R_wp_ = 2.82%) can be used to illustrate that the diffraction pattern was C-type rare earth phase (*Ia-3* (No. 206)) at 1.2 GPa and a lattice constant a = 10.7090 (6) Å. This is the same as the model structure of Gd_2_Ce_2_O_7_ [32]. Figure 2a illustrates the crystal structure of Ho_2_Ce_2_O_7_ oxide, where RE (Ho/Ce) cations occupy 8b and 24d Wyckoff positions with sixfold or eightfold oxygen coordination. Meanwhile, O atoms partly occupy 16c and 48e Wyckoff positions. The cation distribution is characterized by statistically equivalent local oxygen environments for all sites. All cations exhibit uniform spatial distribution and identical local oxygen coordination environments regardless of their chemical identity. Figure 2b shows the possible positions of oxygen vacancies (16c Wyckoff position of x = y = z ≈ 0.391 (2)), where partial occupancy of these sites by oxygen atoms occurs in a randomized spatial arrangement. Under high pressure, the cubic phase of pyrochlores or fluorite transformed into an orthorhombic phase with a defect cotunnite-type structure [33,34]. In_2_O_3_ with a C-type rare earth structure underwent a transformation to a hexagonal corundum-type structure under high pressure [35,36]. The crystal structure of Ho_2_Ce_2_O_7_ exhibited cubic symmetry for pressure values from ambient pressure to 23.8 GPa. At 23.8 GPa, the new diffraction peaks clearly corresponded to the hexagonal phase with space group (*R-3c*), and the lattice parameters were a = 5.7075 (8) Å and c = 16.2623 (6) Å. The phenomena are similar to the research of D. Liu et al. [35]. Above 23.8 GPa, the crystal structure of Ho_2_Ce_2_O_7_ indicates that that cubic phase (*Ia-3*) and hexagonal phase (*R-3c*) coexist. The high-pressure coexistent phase remained up to the highest pressure values we reached; the fitted pattern at 28.8 GPa with R_p_ = 1.45% and R_wp_ = 2.62% is shown in Figure 1c. The determined structural parameters are listed in Table 1.

**Figure 3:**
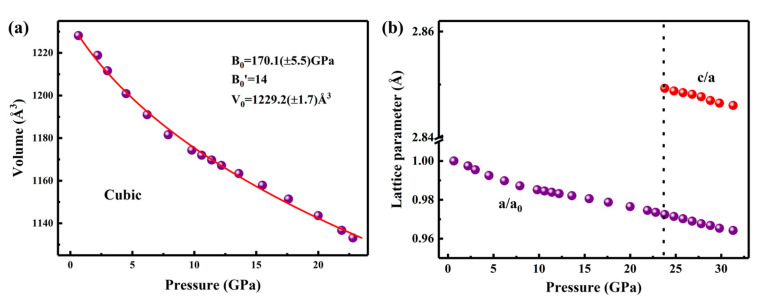
(**a**) Evolution of cell volume at high pressure. The line refers to the fitted third-order Birch−Murnaghan equation of state for the cubic phase. (**b**) The pressure-dependent relative com-pression of a/a_0_ (violet) and c/a (red) for Ho_2_Ce_2_O_7_.

The lattice volume decreased monotonically with increasing pressure as well as a volume collapse occurred at the transition pressure of ~23.8 GPa. As presented in Figure 3a, the P-V diagrams of Ho_2_Ce_2_O_7_ are fitted with a third-order Birch–Murnaghan equation of state (EOS) [37,38]:

(1) $$P=\frac{3}{2}B_{O}\left[{\left(\frac{V_{0}}{V}\right)}^{\frac{7}{3}}-{\left(\frac{V_{0}}{V}\right)}^{\frac{5}{3}}\right]\times \left\{1+\frac{3}{4}\left(B_{o}^{’}-4\right)\times \left[{\left(\frac{V_{0}}{V}\right)}^{\frac{2}{3}}-1\right]\right\}$$

where yields the bulk modulus of B_0_ = 170.1 (±5.5) GPa, initial volume V_0_ = 1229.2 (±1.7) Å^3^ and pressure derivative B_0_′ = 14 for the cubic phase below 23.8 GPa. The large B_0_′ could be attributed to pressure-dependent distortions of the REO_6_ (RE = Ho/Ce) octahedron. The bulk modulus demonstrates slight enhancement compared to the typical fluorite-type La_2_Ce_2_O_7_ (144 GPa) [23]. This may be attributed to the smaller ionic radii of Ho^3+^ and Ce^4+^, which narrows the cubic structure. The bulk modulus B_0_ = 349.1 (±12.9) GPa (with B_0_′ fixed at 4) for the hexagonal phase was estimated from the patterns obtained at high pressure. As shown in Table 2, a substantial volume shrinkage was 700.5 Å^3^ (approximately 62%) at the phase transition pressure. The phase transition was accompanied by chemical bond cleavage between atoms and reconstruction of anion and cation sublattices. Figure 3b illustrates the pressure-dependent relative lattice parameter shrinkage (a/a_0_) for cubic Ho_2_Ce_2_O_7_. The compression ratio (a/a_0_) was reduced by 2.7% in the pressure range from ambient to 23.8 GPa. Notably, in the high-pressure hexagonal phase, the c/a ratio of Ho_2_Ce_2_O_7_ exhibited a gradual decrease from 2.849 to 2.846 as pressure increased from 23.8 to 31.5 GPa. This gradual shrinkage demonstrates that the material experiences slightly greater compressibility along the c-axis compared to the a-axis under hydrostatic compression.

**Figure 4:**
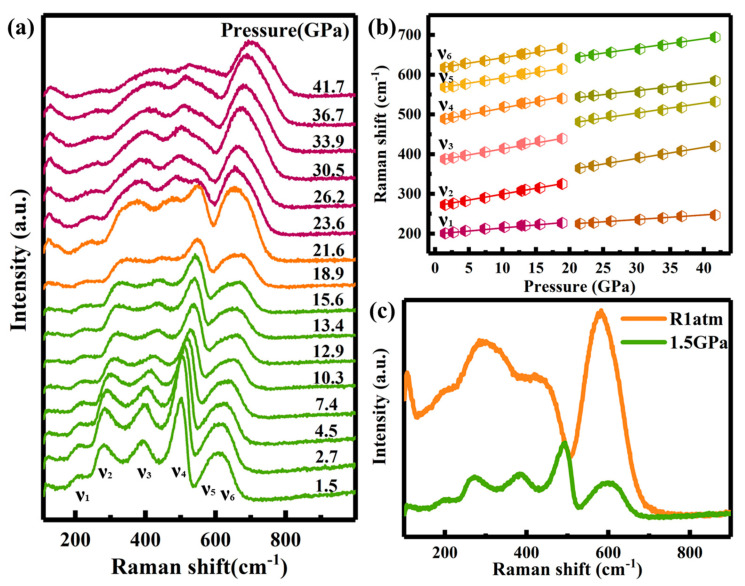
(**a**) Raman spectra of Ho_2_Ce_2_O_7_ at different pressures were recorded during increasing pressure. (**b**) The Raman mode frequency shifts of Ho_2_Ce_2_O_7_ shifting as a function of pressure. (**c**) Raman spectra at 1.5 GPa and full decompression (R1atm) from 41.7 GPa.

To investigate the local structural evolution of Ho_2_Ce_2_O_7_ under high pressure, Raman spectra of Ho_2_Ce_2_O_7_ were systematically conducted up to 41.7 GPa at ambient temperature (Figure 4a). At 1.5 GPa, six vibrational modes were observed (ν_1_: 200 cm^−1^; ν_2_: 272 cm^−1^; ν_3_: 387 cm^−1^; ν_4_: 489 cm^−1^; ν_5_: 569 cm^−1^; ν_6_: 618 cm^−1^), which correspond to the characteristic coordination environments of F-type (CeO_2_) and C-type (Ho_2_O_3_) phases [30]. According to A. Nakajima et al. [39], the band around 600 cm^−1^ split into two distinct peaks at different polarizations in similar compounds. The broad and weak ν_5_ band, which exhibits significant overlap and poor resolution from the ν_6_ peak, originates from intrinsic oxygen vacancies [30]. The high-intensity broad vibrational modes were attributed to structural disorder and the large number of oxygen vacancies in the structure. The ν_4_ mode was assigned to the F_2g_ symmetric vibration mode of the Ce-O bond with Ce eightfold coordination, serving as the fingerprint of the CeO_2_ structure [40,41]. The C-type structure was identified through ν_3_ mode, which resulted from the (A_g_ + F_g_) symmetrical stretching vibration mode of Ho-O with Ho sixfold coordination. The ν_2_, ν_5_ and ν_6_ modes were attributed to the interaction between the oxygen vacancies and the six next-nearest-neighbor oxygen atoms [39]. Additionally, the ν_1_ mode arose from the interaction between the oxygen vacancies and the four nearest-neighbor metal atoms [39].

As showed in Figure 4b, the observed Raman vibrational modes were monotonously shifted to higher wavenumbers with increasing pressure up to 21.6 GPa. The gradual broadening and weakening of Raman vibrational modes may be caused by the change in disorder degree and in polarizability of bonds at pressures above 10.3 GPa. Above 21.6 GPa, the Raman spectra exhibited unambiguous evidence of a change in structural symmetry. The abnormal discontinuities in the Raman vibrational modes and the weakening of the ν_4_ mode indicated the phase transition in Ho_2_Ce_2_O_7_. Above 23.6 GPa, the Raman modes of ν_3_ started to prevail over ν_4_. Meanwhile, the dominance of ν_5_ demonstrated that the interaction between the oxygen vacancies and the six next-nearest-neighbor oxygen atoms became increasingly active under high pressure. These results indicated that ordered cations transition to a disordered state and lead to the variation of the coordination environment. As shown in Figure 4c, the Raman modes of the high-pressure phase were retained after decompression without returning to their original states, demonstrating the irreversible phase transition. These changes in the Raman spectra were consistent with the results of X-ray diffraction data.

## 4. Conclusions

In this study, the crystal structure of Ho_2_Ce_2_O_7_ under high pressure was studied up to 31.5 GPa and 41.7 GPa by means of synchrotron X-ray diffraction and Raman spectroscopy measurements. Rietveld refinements of ADXRD showed that the ambient pressure phase (*Ia-3*) remained stable up to 23.8 GPa at room temperature. At 23.8 GPa, a sluggish structural phase transition occurred and remained uncompleted up to 31.5 GPa. The high-pressure phase is the coexistence of the parent cubic phase (*Ia-3*) and the metastable hexagonal phase (*R-3c*). Above 23.6 GPa, the Raman mode changes showed that ordered cations transition to a disordered state with the variation of the coordination environment. The high-pressure phase is retained after fully releasing pressure, which indicates that the structural phase transition was irreversible.

## Figures and Tables

**Table 1:** The refined atomic coordinates of the cubic phase and the high-pressure phase of Ho_2_Ce_2_O_7_ at 1.2 and 28.8 GPa, respectively.

Compounds	Ho_2_Ce_2_O_7_ (1.2 GPa)	Ho_2_Ce_2_O_7_ (28.8 GPa)	
Crystal System	Cubic	Cubic	Hexagonal
Space group	*Ia-3* (No. 206)	*Ia-3* (No. 206)	*R-3c* (No. 167)
a/Å	10.7090 (6)	10.3529 (2)	5.6984 (12)
b/Å	10.7090 (6)	10.3529 (2)	5.6984 (12)
c/Å	10.7090 (6)	10.3529 (2)	16.2232 (09)
R_p_	1.44%	1.45%	
R_wp_	2.82%	2.62%	
R_exp_	3.22%	3.12%	
χ	0.88	0.84	
Atoms	Wyckoff (x y z)	Wyckoff (x y z)	Wyckoff (x y z)
Ho1/Ce1	24d (−0.0107 (3) 0 0.25)	24d (−0.0043 (6) 0 0.25)	12c (0 0 0.872 (9))
Ho2/Ce2	8b (0.25 0.25 0.25)	8b (0.25 0.25 0.25)	
O (1)	48e (0.381 (5) 0.138 (8) 0.362 (2))	48e (0.368 (8) 0.161 (6) 0.392 (9))	18e (0.306 (2) 0 0.25)
O (2)	16c (0.391 (2) 0.391 (2) 0.391 (2))	16c (0.484 (9) 0.484 (9) 0.484 (9))	

Note: The numbers in parentheses are the estimated standard deviations in the units of the accompanying digit.

**Table 2:** Unit cell parameters and volumes of Ho_2_Ce_2_O_7_ under varying pressure conditions. Above 23.8 GPa, the lattice constants of the cubic phase and the hexagonal phase are given because they both exist in significant amounts.

Pressure (GPa)	Symmetry	a(Å)	c(Å)	V(Å^3^)
1.2	Cubic	10.7092 (6)		1228.1 (11)
3	Cubic	10.6607 (3)		1211.6 (5)
4.5	Cubic	10.6291 (4)		1200.8 (2)
6.2	Cubic	10.5998 (8)		1190.9 (07)
7.9	Cubic	10.5718 (7)		1181.5 (3)
9.8	Cubic	10.5503 (2)		1174.3 (5)
12.2	Cubic	10.5287 (09)		1167.1 (4)
15.5	Cubic	10.5007 (2)		1157.8 (3)
17.6	Cubic	10.4812 (8)		1151.4 (6)
20.1	Cubic	10.4574 (5)		1143.6 (8)
23.8	Cubic	10.4139 (6)		1129.5 (5)
	Hexagonal	5.7075 (8)	16.2623 (6)	429.1 (2)
26.4	Cubic	10.3770 (3)		1117.4 (2)
	Hexagonal	5.7012 (2)	16.2381 (5)	426.8 (14)
28.8	Cubic	10.3529 (07)		1109.6 (3)
	Hexagonal	5.6984 (12)	16.2232 (09)	424.1 (11)
31.5	Cubic	10.3260 (9)		1101.1 (4)
	Hexagonal	5.6941 (4)	16.2059 (06)	422.3 (2)

Note: The numbers in parentheses are the estimated standard deviations in units of the last digit.

## Data Availability

The original contributions presented in this study are included in the article. Further inquiries can be directed to the corresponding authors.
